# STAT3 dominant negative Hyper-IgE syndrome: A patient report with actionable genomic findings

**DOI:** 10.1016/j.ejmg.2026.105077

**Published:** 2026-04-13

**Authors:** Aislinn S. Bloom, Laura M. Amendola, Nadjalisse Reynolds-Lallement, Amanda Urban, Alexandra F. Freeman, Magdalena A. Walkiewicz, Morgan N. Similuk

**Affiliations:** a Division of Intramural Research, National Institute of Allergy and Infectious Diseases (NIAID), National Institutes of Health (NIH), 10 Center Drive, Bethesda, MD, 20892, USA; b Clinical Research Directorate, Frederick National Laboratory for Cancer Research, National Institutes of Health (NIH), 10 Center Drive, Bethesda, MD, 20892, USA; c Laboratory of Clinical Immunology and Microbiology, National Institute of Allergy and Infectious Diseases, National Institutes of Health (NIH), 10 Center Drive, Bethesda, MD, 20892, USA

**Keywords:** Genomics, Pharmacogenomics, Secondary finding, STAT3 dominant negative hyper-IgE syndrome, Familial hypercholesterolemia

## Abstract

Over the last two decades, diagnostic genetic testing for inborn errors of immunity has primarily relied on gene panel-based approaches organized by phenotype. In this report, we describe a patient with a clinical and molecular diagnosis of STAT3 dominant negative hyper-IgE syndrome referred to the National Institutes of Health for further evaluation including genome sequencing. Genome sequencing analysis included primary, secondary, and pharmacogenomic findings. This analysis confirmed the primary molecular diagnosis of STAT3 dominant negative hyper-IgE syndrome and found a pathogenic variant in the *LDLR* gene associated with familial hypercholesterolemia, which led to the identification of borderline high LDL-C levels in the patient. Additionally, this analysis identified pharmacogenomic genotypes associated with suboptimal therapeutic effects for the frontline treatments of the patient’s conditions. Specifically, the patient was found to be a rapid metabolizer of voriconazole, a treatment for severe fungal infections, and to have an increased risk for myopathy induced by taking statins, the most common treatment for familial hypercholesterolemia. This case underscores the potential clinical utility of comprehensive genomic evaluation for patients with rare diseases. The untargeted nature of genome sequencing and broad range of potential findings can inform treatment decisions, shorten the diagnostic odyssey, and enable a more personalized approach to patient care.

## 1. Introduction

STAT3 dominant negative (DN) hyper-IgE syndrome (STAT3DN-HIES; Job’s Syndrome), is a rare inborn error of immunity (IEI) characterized by multisystem infectious and non-infectious features including skin and lung infections, dermatitis, scoliosis, minimal trauma fractures, and elevated serum IgE (Tsilifis et al., 2021). Affected individuals usually present with symptoms in the early weeks of life. STAT3DN-HIES is caused by pathogenic variants in the *STAT3* gene that result in DN STAT3 protein function and disrupt the JAK/STAT signaling pathway. STAT3DN-HIES is inherited in an autosomal dominant pattern and *de novo* cases are frequent (Tsilifis et al., 2021; Hsu et al., 1993). Penetrance of STAT3DN-HIES appears to be complete, though there can be variable expressivity of phenotypes even between family members with the same pathogenic variant (Tsilifis et al., 2021).

Panel-based genetic testing of immune genes is routinely integrated into diagnostic evaluations for immunology patients, particularly within pediatrics (von Hardenberg et al., 2024). Such approaches typically include sequencing dozens to hundreds of IEI-associated genes. While panel testing can be a cost-effective approach to molecular diagnostics, it has limitations in detecting variants outside the target region and some complex variant types. Thus, more comprehensive genetic testing such as exome (ES) or genome sequencing (GS) that includes the analysis of all annotated variants can increase the diagnostic yield for IEI and can also provide additional medically actionable information.

Recent literature suggests higher diagnostic yield for rare disease patients when utilizing comprehensive genetic testing such as ES or GS versus panel-based testing. A study led by Similuk et al. reported findings from ES in 1000 probands with suspected IEI, where 10% of individuals with a known primary molecular diagnosis were found to have an additional reportable finding, including but not limited to medically actionable secondary findings (SF) (Similuk et al., 2022). A second study by Thaventhiran et al., performed GS in a cohort of 1318 participants with sporadic primary immunodeficiency (PID), leading to overall diagnostic yield of 17% (Thaventhiran et al., 2020). Taken together, these studies highlight the potential value of providing broad genetic testing via ES and GS to patients with rare IEI.

The identification and return of pharmacogenomic (PGx) variants from GS can also contribute to more comprehensive personalized care. Most individuals who have PGx testing for actionable PGx genotypes have at least one finding associated with reduced efficacy or increased toxicity of certain therapeutic agents, including some which are widely prescribed (e.g., statins, proton pump inhibitors) (Cousin et al., 2017). Identification of these alleles can improve medications therapeutic efficacy, safety, and compliance (Jensson et al., 2023).

Here, we describe a patient with a clinical and molecular diagnosis of STAT3DN-HIES who underwent GS, which identified additional actionable findings. This case highlights the potential impact of a comprehensive genomic workup, including the return of SFs and PGx results, in guiding clinical decisions for patients with IEI. By demonstrating the utility of GS in identifying both disease-causing variants and additional clinically relevant information, this report highlights the potential of integrating GS into routine clinical care for patients with rare diseases, including IEIs, offering a more personalized and effective approach to diagnosis and treatment.

## 2. Case presentation

### 2.1. Clinical history

A 4-year-old female born from an uncomplicated pregnancy, presented at the National Institutes of Health (NIH) for clinical research evaluation based on her clinical and molecular diagnosis of STAT3DN-HIES.

The patient initially came to medical attention at four months of age, with a newborn rash and bacterial pneumonia with a pleural effusion, requiring a chest tube, intravenous vancomycin, and a greater than two-week hospital stay. The patient subsequently developed eczema, located on her forehead, chest, and back; cold abscesses of the head and neck that required incision and drainage; and chronic thrush.

At age two, the physical examination noted her weight to be 11.4kg (Z-score: −1.13), height 87.9cm, and head circumference 47cm (10th percentile)(Rollins et al., 2010). The patient had multiple caries and abscesses, that required extraction and capping of her remaining teeth. Musculoskeletal abnormalities, including pectus excavatum, frontal bossing, high palate, and joint hyperextensibility were noted. The patient had a radial fracture from a fall while playing. The patient required another hospitalization at the age of two due to severe right lower lobe pneumonia complicated by empyema. Outside labs consistently noted elevated IgE serum levels up to 867 kU/L.

**Fig. 1.**
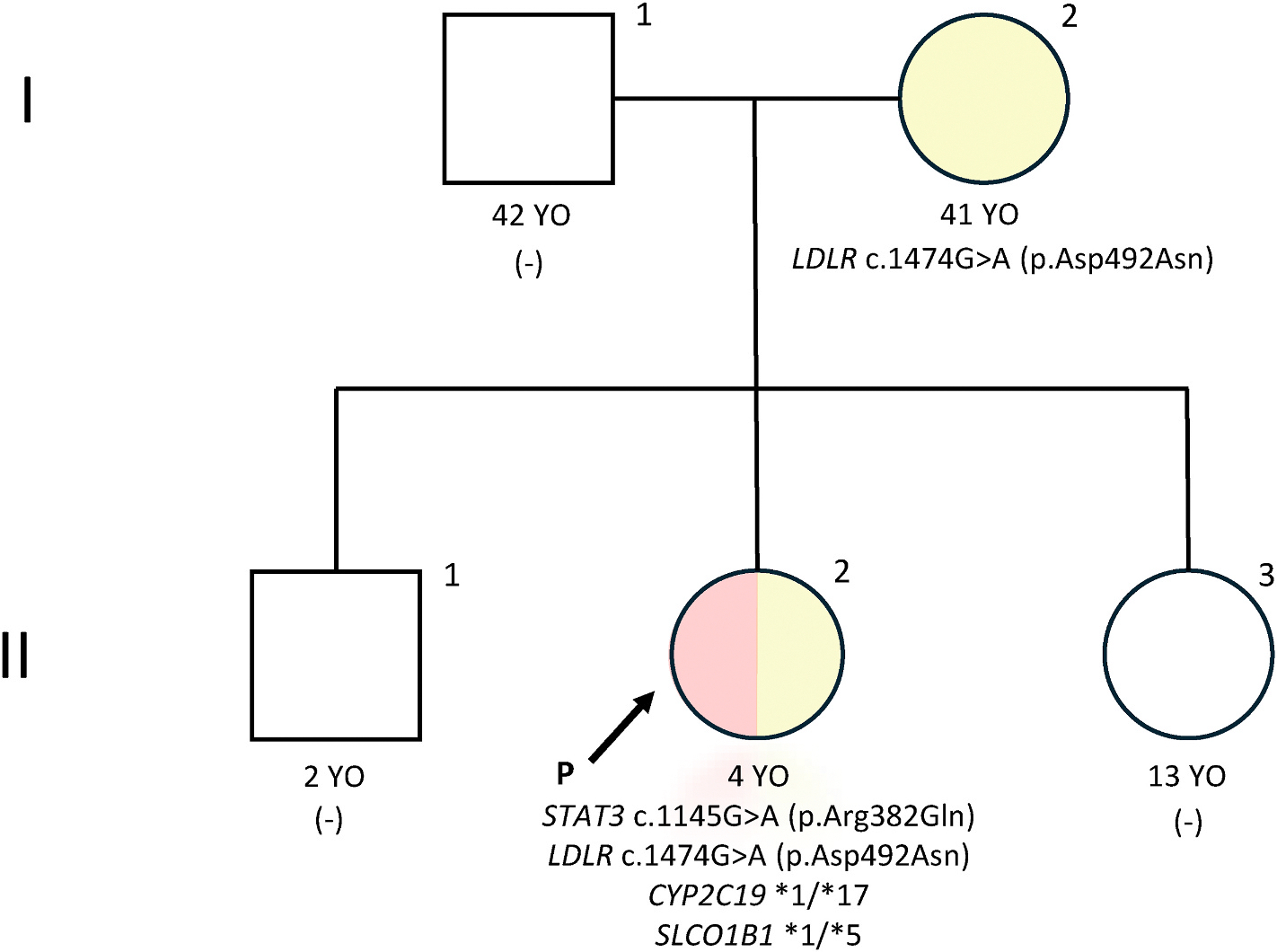
Pedigree with Genotypes. Pedigree of this proband (II-2). Square, male; circle, female; P and arrow, proband. A light-red fill indicates that an individual has STAT3DN-HIES. A light-yellow fill indicates an individual has familial hypercholesteremia. (−) indicates an individual has not received genetic testing. The *STAT3* pathogenic variant c.1145G > A (p.Arg382Gln) is present in the proband, but not her mother. The *LDLR* pathogenic variant c.1474G > A (p.Asp492Asn) is present in both proband and mother. The proband has PGx alleles of CYP2C19*1/*17 (rapid metabolism of voriconazole) and SLCO1B1*1/*5 (risk for statin-induced myopathy).

The patient was born from non-consanguineous parents. Her father has a significant history of eczema and asthma, and her mother reported a two-year period of developing multiple abscesses, from ages 9 to 11 years. The patient’s older sister and younger brother both have no significant medical history and no other family members are similarly affected. The pedigree taken on evaluation at the NIH is shown in Fig. 1.

### 2.2. Genetic testing results

Prior to her evaluation at the NIH, this patient’s clinical diagnosis of STAT3DN-HIES had been molecularly confirmed through a genetic testing panel of 429 IEI-associated genes which identified a heterozygous *STAT3* pathogenic missense variant (c.1145G > A, p.Arg382Gln).

Based on this diagnosis, the patient was referred to the NIH, National Institute of Allergy and Infectious Diseases (NIAID), Centralized Sequencing Program for GS to support her primary NIH research program that is investigating genomic contributions to the presentation, natural history, and management of patients with STAT3DN-HIES. The GS analysis identified the previously detected heterozygous c.1145G > A (p. Arg382Gln) pathogenic variant in *STAT3*. In addition, a SF analysis detected a heterozygous c.1474G > A, (p.Asp492Asn) pathogenic variant in the *LDLR* gene associated with familial hypercholesterolemia. PGx analysis detected the *CYP2C19* *1/*17 genotype associated with rapid metabolism of voriconazole and increased risk for therapeutic failure necessitating careful dose titration; as well as the *SLCO1B1* *1/*5 genotype, which is associated with decreased function of SLCO1B1, increasing the risk for toxicity, specifically statin-induced myopathy, and necessitating potential dose adjustment. Subsequent follow up Sanger sequencing studies showed that the patient’s mother was negative for the *STAT3* pathogenic variant and heterozygous for the *LDLR* pathogenic variant. PGx testing was not done on the patient’s mother. The patient’s father was not tested.

### 2.3. Ongoing management

The patient continues to be monitored at the NIH clinical center for STAT3DN-HIES. Physical examination noted her weight to be 14.2kg (Z-score: −1.66), height 103.6cm (Z-score: −0.5), and BMI 13.2kg/m^2^ (Z-score: −2.11, indicating moderate malnutrition). In her most recent evaluation, the patient presented with chronic paronychia on the right thumb, oral candidiasis, mild intertrigo, pruritus, and no respiratory infections or fevers. Fluconazole was started for the candidiasis. Recent labs indicated IgE serum levels of 862 IU/mL. Low-density lipoprotein (LDL) cholesterol levels were ordered based on the *LDLR* pathogenic variant finding and were measured at 170mg/dL, which is in the borderline high range for pediatric patients. The patient’s current dietary recommendations include a soft diet to accommodate lack of teeth, nutritional supplements, one multivitamin chewable tablet daily, and limiting intake of saturated fat and cholesterol.

Care of STAT3DN-HIES is supportive with the use of antimicrobial agents to prevent infections. The patient is taking Sulfamethoxazole/Trimethoprim to prevent staphylococcal infections, fluconazole given her history of thrush and onychomycosis, and intravenous immunoglobulin (IVIG) monthly. No changes in medications had been made at the time of this case report based on the PGx findings.

## 3. Discussion

We describe a case of a 4-year-old female who presented at the NIH with a prior clinical and molecular diagnosis of STAT3DN-HIES. A comprehensive genomic evaluation, through GS, identified the previously reported pathogenic variant in *STAT3*, a SF of a heterozygous pathogenic variant in *LDLR* associated with familial hypercholesterolemia, and PGx genotypes indicating suboptimal therapeutic effect of treatments for both STAT3DN-HIES and familial hypercholesterolemia (*CYP2C19* *1/*17 and *SLCO1B1* *1/*5, respectively). This case highlights how comprehensive genomic evaluation identified actionable health information that impacted both the patient’s immediate medical management and potential future care for her and her family.

These findings are consistent with prior cohort studies demonstrating that comprehensive sequencing in IEI frequently identifies clinically actionable results beyond the primary diagnosis. In a large ES cohort of individuals with suspected IEI, additional reportable findings were identified in approximately 10% of those with an established molecular diagnosis, including medically actionable secondary findings (Similuk et al., 2022). Similarly, GS in a sporadic PID cohort achieved a diagnostic yield of 17%, including pathogenic variants in known disease genes and modifier loci (Thaventhiran et al., 2020). Together, these data reinforce that broad genomic approaches can uncover clinically relevant information that would not be captured through targeted testing alone, as illustrated in this case.

### 3.1. STAT3DN-HIES and pulmonary management

STAT3DN-HIES manifests with both infectious and non-infectious complications. Severe pulmonary infections, particularly recurrent pneumonia, are a primary concern, with approximately 80% of affected individuals experiencing recurrent episodes that can lead to pleural effusions and parenchymal disease, including bronchiectasis and pneumatoceles (Tsilifis et al., 2021). Both bacterial and fungal pulmonary infections are associated with increased mortality and are common as the disease progresses. Given this patient’s history of two severe pneumonias complicated by pleural effusion and empyema by age four years, her current management is focused on prevention, such as with prophylactic antimicrobials and IVIG, and aggressive treatment to reduce the risk of developing parenchymal lung disease.

### 3.2. Pharmacogenetic insights into antifungal therapy

Voriconazole is a common antifungal used to treat *Aspergillus* in immunocompromised patients, including those with IEI such as STAT3DN-HIES (Clinical Pharmacogenetics Implementation Consortium). This patient’s PGx analysis identified that the patient has the *CYP2C19* *1/*17 genotype which leads to rapid metabolism of voriconazole and decreases the probability of attaining a therapeutic dose (Clinical Pharmacogenetics Implementation Consortium). The Clinical Pharmacogenetic Implementation Consortium (CPIC) recommends that pediatric patients with this rapid metabolism phenotype undergo diligent therapeutic drug monitoring starting at the standard dose and adjusting as needed. CPIC also recommends that providers consider selecting an alternative therapy due to the amount of time that is needed to identify an effective therapeutic drug dose for patients in critical states (Clinical Pharmacogenetics Implementation Consortium). Achieving effective therapeutic levels as soon as possible when *Aspergillus*-related pulmonary infections occur in patients with STAT3DN-HIES is needed to mitigate prolonged illness and avoid a potentially fatal outcome. Given the importance of timely treatment to reduce mortality from Aspergillus infections, the knowledge of this patient’s *CYP2C19* genotype can enable a more personalized and potentially life-saving approach to therapy.

Of note, genome sequencing outperforms exome sequencing for PGx testing because it captures non-coding pharmacogenomic variants that exome sequencing misses. For example, the patient’s *CYP2C19* *1/*17 genotype lies in the promoter region and would likely have been miscalled as *1/*1 by exome sequencing, limiting treatment personalization. As cost differences narrow, genome sequencing is expected to become more common. It also improves detection of repeat expansions, structural variants, and other non-coding changes, though higher cost currently limits access.

### 3.3. Familial hypercholesterolemia (FH) and cardiovascular risk

FH is an inherited disorder characterized by elevated LDL cholesterol levels and an increased risk of cardiovascular disease (CVD) (Harada-et al., 2023). The patient’s GS identified a pathogenic *LDLR* variant, consistent with FH. Pediatric patients with FH are recommended to maintain their LDL cholesterol levels below 140 mg/dL, and if levels exceed 180 mg/dL, statin therapy is recommended starting at age 8 or 10 (Cooper-DeHoff et al., 2022;Harada-Shiba et al., 2023). This patient’s LDL cholesterol level was measured at 170 mg/dL, which is borderline high, and will require ongoing monitoring and dietary modification. Early identification through GS allowed for the timely detection of this risk, which could have gone unnoticed without comprehensive evaluation. Additionally, cascade testing of the patient’s mother detected the same *LDLR* variant which provided the opportunity for her mother to seek counseling and treatment, and highlights the utility of familial testing. Cascade testing for other family members would provide further actionable health information for the entire family.

### 3.4. Pharmacogenetics and statin therapy

This patient’s PGx genotype of *SLCO1B1* *1/*5 is associated with an increased risk for statin-induced myopathy, which is a concern for patients requiring statin therapy for FH (Cooper-et al., 2022). Although PGx guidelines for pediatric statin therapy are still evolving, CPIC offers dosing recommendations for adults that will be applicable as the patient ages. Her PGx results can inform clinician decision making to minimize the adverse effects from statin therapy and therefore optimize her treatment adherence and efficacy.

### 3.5. Barriers to healthcare access

While comprehensive GS has the potential to detect a range of actionable health risks, patients may face barriers to following up on medical recommendations. At the time of this publication, the family described in this case report has not been able to follow up on the FH secondary finding due to multiple hurdles in accessing healthcare, including finances, a lapse in employment/health insurance, relocation, additional illness in the family, immigration status, and the time burden of coordinating specialty care for multiple rare, chronic conditions. Emphasis on appropriate referrals to specialists and resources for families during genetic counseling disclosures are vital to facilitating ideal health outcomes.

Additionally, this case report is an illustration in the United States for the clinical utility of opportunistic screening (SFs and PGx) through comprehensive genetic testing. Historically, the American College of Medical Genetics (ACMG) and the European Society of Human Genetics (ESHG) have taken different approaches on opportunistic screening (de Wert et al., 2021). ESHG recommends a cautious approach to genetic testing, limiting opportunistic screening to focus on clear clinical indications, meanwhile, ACMG recommends a broader approach promoting opportunistic screening regardless of clinical indication. ESHG recommends a more cautious approach to opportunistic screening due to the benefits-and-risks balance, publicly-funded healthcare limitations, and ethical concerns, especially in regards to the return of adult-onset conditions to minors (de Wert et al., 2021). This case report contributes to the literature on the different approaches recommended by ACMG versus ESHG for opportunistic screening, eliciting further discussions on the clinical utility of these results.

## 4. Conclusion

**Fig. 2.**
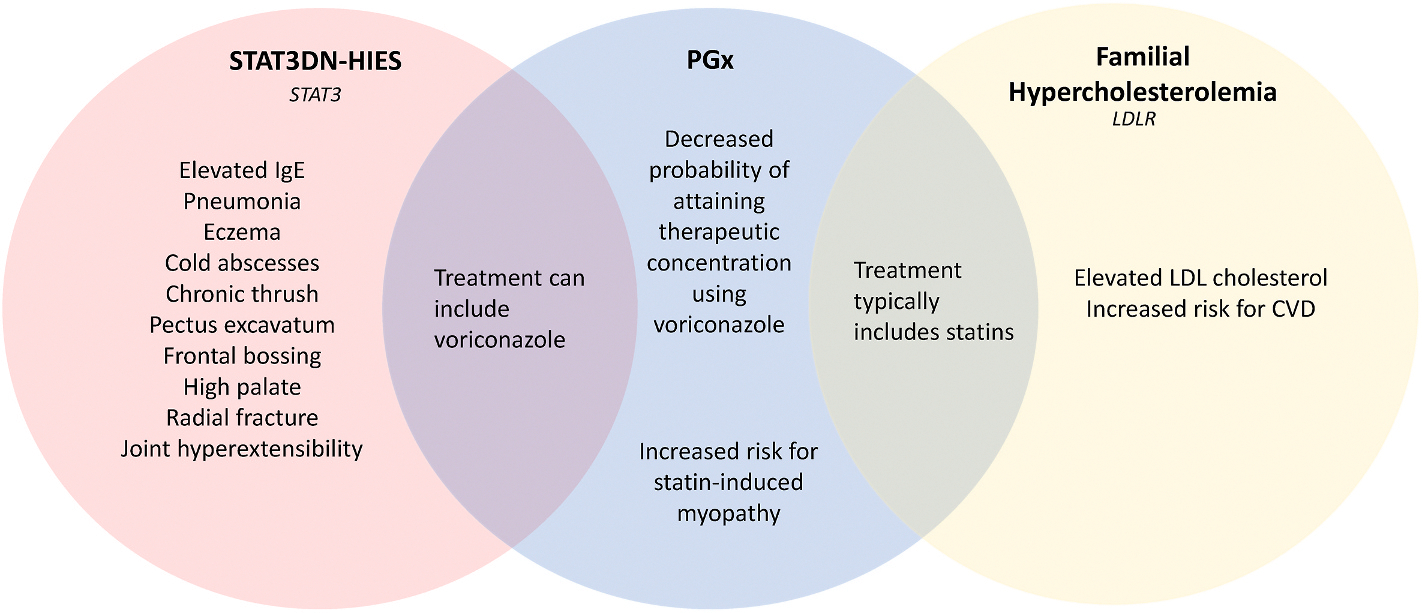
Venn Diagram with Genotype-Phenotype Summary. Illustrates the phenotypes associated with the proband’s diagnostic and secondary findings and displays the potential impact of her PGx findings on the treatment for both STAT3DN-HIES and familial hypercholesterolemia. CVD stands for cardiovascular disease.

This comprehensive genomic evaluation identified a secondary finding of FH and two clinically relevant PGx genotypes, in addition to identifying the previously known primary diagnosis of STAT3DN-HIES [Fig. 2]. The patient’s prior targeted genetic testing panel did not capture the breadth of clinically actionable health information available through GS in this case. This case underscores the value of comprehensive GS in patients with rare diseases, highlighting how the identification of SFs and PGx variants can provide actionable insights that directly influence medical management. Pre-emptive knowledge of such information not only reduces trial-and-error treatment approaches but also facilitates a more personalized and effective care plan. This case exemplifies the potential of genomics to transform patient care, offering a bold vision for the future of precision medicine.

## Data Availability

Data will be made available on request.
